# Control of Structural and Magnetic Properties of Polycrystalline Co_2_FeGe Films via Deposition and Annealing Temperatures

**DOI:** 10.3390/nano11051229

**Published:** 2021-05-07

**Authors:** Andrii Vovk, Sergey A. Bunyaev, Pavel Štrichovanec, Nikolay R. Vovk, Bogdan Postolnyi, Arlete Apolinario, José Ángel Pardo, Pedro Antonio Algarabel, Gleb N. Kakazei, João Pedro Araujo

**Affiliations:** 1 Departamento de Física e Astronomia, Institute of Physics for Advanced Materials, Nanotechnology and Photonics (IFIMUP), Universidade do Porto, 4169-007 Porto, Portugal; sergiy.bunyayev@gmail.com (S.A.B.); nikolayvovk94@gmail.com (N.R.V.); b.postolnyi@gmail.com (B.P.); arlete.apolinario@fc.up.pt (A.A.); gleb.kakazei@fc.up.pt (G.N.K.); jearaujo@fc.up.pt (J.P.A.); 2 Instituto de Nanociencia y Materiales de Aragón, Campus Río Ebro, Universidad de Zaragoza—CSIC, 50018 Zaragoza, Spain; stricho@unizar.es (P.Š.); jpardo@unizar.es (J.Á.P.); 3 Department of Nanoelectronics and Surface Modification, Sumy State University, 40007 Sumy, Ukraine; 4 Departamento de Ciencia y Tecnología de Materiales y Fluidos, Universidad de Zaragoza, 50018 Zaragoza, Spain; 5 Instituto de Nanociencia y Materiales de Aragón, Campus San Francisco, Universidad de Zaragoza—CSIC, 50009 Zaragoza, Spain; algarabe@unizar.es; 6 Departamento de Física de la Materia Condensada, Universidad de Zaragoza, 50009 Zaragoza, Spain

**Keywords:** thin films, Heusler alloys, magnetostatic properties, Ferromagnetic resonance

## Abstract

Thin polycrystalline Co_2_FeGe films with composition close to stoichiometry have been fabricated using magnetron co-sputtering technique. Effects of substrate temperature (T_S_) and post-deposition annealing (T_a_) on structure, static and dynamic magnetic properties were systematically studied. It is shown that elevated T_S_ (T_a_) promote formation of ordered L2_1_ crystal structure. Variation of T_S_ (T_a_) allow modification of magnetic properties in a broad range. Saturation magnetization ~920 emu/cm^3^ and low magnetization damping parameter α ~ 0.004 were achieved for T_S_ = 573 K. This in combination with soft ferromagnetic properties (coercivity below 6 Oe) makes the films attractive candidates for spin-transfer torque and magnonic devices.

## 1. Introduction

Full-Heusler alloys (FHA) are ternary intermetallic compounds of composition X_2_YZ, where X and Y are transition metals and Z is a main-group *sp*—element. Recently they attracted a lot of attention due to unique physical properties suitable for advanced applications (See e.g., [1] and references therein). Some FHAs demonstrate half-metallic ferromagnetic (HMF) properties, that are crucial for spintronics. The term HMF [2] is used to describe a material with strong asymmetry in spin-split band structure. This means that the majority spin band has metallic characteristics (i.e., Fermi level (ε_F_) is located within the band with finite density of states (DOS)) but for the minority one, ε_F_ is situated in a band gap (DOS = 0). This leads to 100% spin polarization of conduction electrons. Half metallicity can be used to enhance key properties of magnetic field sensors based on Giant or Tunneling magnetoresistance effect (GMR/TMR), magnetic random-access memory (MRAM) and spin-transfer torque (STT) devices [3]. Several Co_2_-based FHA were found to be HMF materials and studied intensively [2,4,5,6,7,8]. Another attractive HMF feature (particularly for magnonic applications [9]) is low values of Gilbert damping parameter (α) [10]. Previously very low α were reported for epitaxial Co_2_FeAl (α ~ 0.001) [11], Co_2_MnSi (α ~ 0.0004–0.0025) [12,13] and Co_2_FeGe (α ~ 0.0025) [5] FHA films. It was shown [14] that α is proportional to the product ξ^2^D(ε_F_), where ξ is the spin-orbit coupling parameter and D(ε_F_) is the density of states at Fermi level. While ξ is already low for FHA due to small orbital moments [15], vanishing values of D(ε_F_) in one of the spins channels of HMF reduce α even further. According to the model proposed in [16], one should increase spin polarization and reduce α, saturation magnetization (*M_S_*) and exchange stiffness constant (*A*) of a ferromagnetic material to achieve a decrease in the critical current (*J_c_*) and minimize the power consumption required for the STT switching. Thus, FHA with high spin polarization and moderate values of *M_S_* and *A* comparing to ferromagnetic metals like Fe and Co are attractive candidates for STT applications. On the other hand, a large value of α is needed to improve thermal stability in current-perpendicular-to-plane giant magnetoresistance (GMR) read sensors [17] Physical properties of FHA can be effectively tuned in a wide range by changing atomic ordering, microstructure and composition [1,2,4,17,18,19,20,21]. Thus, FHA films are considered as attractive candidates for different multifunctional applications. It was shown in [5,17,19,22,23] that substrate temperature (T_S_) and post-deposition annealing (T_a_) are powerful tools to control the properties of FHA films. However, so far there is no universal law for thermal treatment conditions. They should be chosen individually depending on composition on the film and preparation strategy.

In this work Co_2_FeGe polycrystalline films were fabricated using magnetron co-sputtering at different conditions. Here we report on the control of the structure, static and dynamic magnetic properties via deposition temperature and post-deposition annealing. We found that damping can be tuned in a broad range by adjusting deposition temperature. The values of parameters derived from the experiment were analyzed and compared to those known from the literature.

## 2. Materials and Methods

Heusler alloy films of composition Co_2_FeGe and thickness ~60 nm were grown onto 25 × 25 mm^2^ Corning Glass substrates using Orion-5 deposition system (AJA International Co., Scituate, MA, USA). The system was operated under full computer control allowing programing the deposition sequences and reproduction of the technological conditions with high accuracy. Co-sputtering from two independent sources technique was used for sample preparation. Previously it was shown that this technique is very effective for preparation of different nanostructures [24,25] and Co_2_FeGe thin films of various composition [6,20,21]. Deposition was performed from two independent direct current 2′′ magnetron sputter sources loaded with high purity (better than 99.99 at. %) Co_2_Fe alloy and Ge targets (Testbourne Ltd., Basingstoke, UK). Base pressure was below 2 × 10^−7^ Torr and the depositions were carried out at 3 mTorr of Ar. The deposition rates were determined from thickness measurements of the reference films. The values of 7.7 nm/min for Co_2_Fe and 5.1 nm/min for Ge were selected to ensure stoichiometric composition of the resulting alloy. During film preparation, the rates were kept constant via the control of the deposition current. The substrates were mounted on a rotating sample holder (25 RPM) that was positioned at 120 mm distance from the deposition sources to provide uniformity of the films thickness and composition. Films were deposited at room temperature (RT), and elevated temperatures T_S_ = 573 K and T_S_ = 773 K. After deposition the samples were kept 4 h in the chamber in the 3 mTorr Ar atmosphere for cooling. To study the effect of annealing the films were deposited at T_S_ = RT and annealed in-situ in 3 mTorr Ar flow at T_a_ = 573 K and T_a_ = 773 K for 1 h. The cooling sequence was the same as for the films deposited at the elevated T_S_.

The composition of the films was checked by Energy Dispersive X-Ray analysis using FEI Quanta 400FEG Field Emission SEM/EDAX-PEGASUS X4M (FEI Co., Hillsboro, OR, USA). X-ray investigations were carried out using Rigaku SmartLab X-ray diffractometer (Rigaku Co., Tokyo, Japan) equipped with a primary Ge crystal monochromator (Cu-Kα radiation). The crystal structure of the films was investigated using grazing incidence X-ray diffraction (GIXRD) in parallel beam geometry with 0.6° incidence angle (right above the maximum critical angle among all the samples, 0.37°). X-ray reflectivity (XRR) measurements were also performed to evaluate thickness, surface oxidation and roughness of the films. To extract abovementioned parameters the experimental XRR profiles were fitted using LEPTOS software version 2.02 (Bruker AXS GmbH, Karlsruhe, Germany).

Static magnetic characterization of the films was performed using Quantum Design MPMS superconducting quantum interference device magnetometer (Quantum Design Inc., San Diego, CA, USA). Magnetic hysteresis loops were measured at RT and in magnetic field *H* up to 500 Oe applied in the film plane. All spurious instrumental effects and those associated with paramagnetic substrate contribution [26,27] were carefully accounted for and subtracted from the initial magnetization curves.

Ferromagnetic resonance (FMR) measurements were carried out at room temperature using a coplanar waveguide (CPW) connected to a vector network analyzer (VNA) Anritsu 37247D (Anritsu Co. Inc., Atsugi, Japan). A dc magnetic field was applied in film plane perpendicularly to the direction of the magnetic component of the microwave field. The samples were placed film down on the CPW, and the complex S_21_ parameter was measured as a function of the external magnetic field over a frequency range up to 20 GHz. The FMR frequency *f* and resonance linewidth Δ*H* were extracted from the raw data as described in [28,29].

## 3. Results and Discussion

### 3.1. Structural Properties

The composition of the films under investigation (in atomic percent) checked by EDAX varies from Co_48_Fe_23_Ge_29_ to Co_48_Fe_21_Ge_31_. This slightly differs from targeted stoichiometry Co_50_Fe_25_Ge_25_.

**Figure 1:**
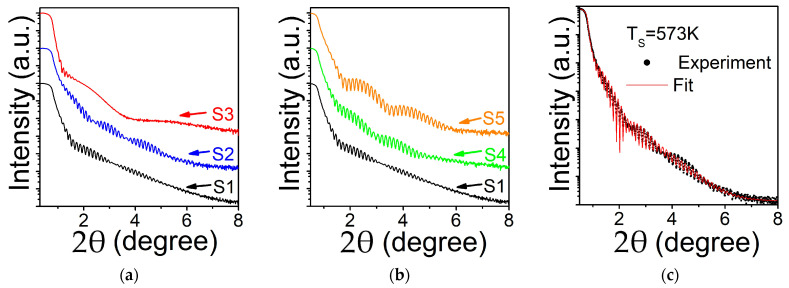
X-ray reflectivity for Co_2_FeGe films: (**a**) deposited at T_S_ = RT (S1), T_S_ = 573 K (S2), T_S_ = 773 K (S3); (**b**) deposited at RT and annealed for 1 h at T_a_ = 573 K (S4), T_a_ = 773 K (S5); (**c**) fit of the experimental XRR spectrum for the film deposited at T_S_ = 573 K. Fitting parameters are summarized in Table 1.

Figure 1 shows XRR of the films deposited at different T_S_ (a) and deposited at RT and annealed at T_a_ (b).

Films deposited at T_S_ = RT and 573 K exhibit a periodic pattern in a wide diffraction angle, suggesting a flat layer structure, while the one at T_S_ = 773 K shows a fast drop and decay of Kiessig oscillations, indicating a rough surface. In addition, there is a visible two period oscillations around 2θ ≈ 3–3.5° (T_S_ = RT) and 2θ ≈ 2.25–2.5° and 2θ ≈ 3.5–3.75° (T_S_ = 573 K), which can be interpreted as due to the formation of a thin oxide surface layer. The XRR data for annealed films retain periodic pattern for all T_a_ but still show two period oscillations. Fit of the experimental data (Figure 1c) allows estimation of thickness, roughness and density. Film and native oxide densities were set as free parameters since the exact chemical composition of the oxide is not known, and the density of the film can be different from the bulk. Bulk densities for Co_3_O_4_ oxide (6.11 g/cm^3^) and Co_2_FeGe alloy (8.66 g/cm^3^) were chosen as initial values. The best fitting parameters are presented in Table 1. For all the films the estimated thickness of Co_2_FeGe is within 58 nm ± 3 nm range that corresponds well to targeted 60 nm value. The thickness of native oxide layer was evaluated between 2 and 5 nm with the density ~6.5 g/cm^3^ for all the samples. The estimated densities of the films deposited at elevated T_S_ are close to bulk values. However, films deposited at T_S_ = RT and those annealed afterwards at T_a_ = 573 K show lower density (as reflected by a slightly smaller critical angle). The former case can be explained as due to smaller diffusion rates of adatoms during deposition, while the latter because relatively low T_a_ does not provide conditions for efficient re-crystallization. Surface roughness for the films deposited at T_S_ = RT and for annealed at different T_a_ was determined to be ~0.63 nm independently of the annealing temperature used in this study. The lowest surface roughness (0.47 nm) was found for the film deposited at T_S_ = 573 K while for T_S_ = 773 K it is much higher (~2 nm). Low surface roughness is a key factor for preparation of the electrodes for TMR tunnel junctions [30]. From this aspect the films deposited at T_S_ = 573 K are the best candidates.

**Figure 2:**
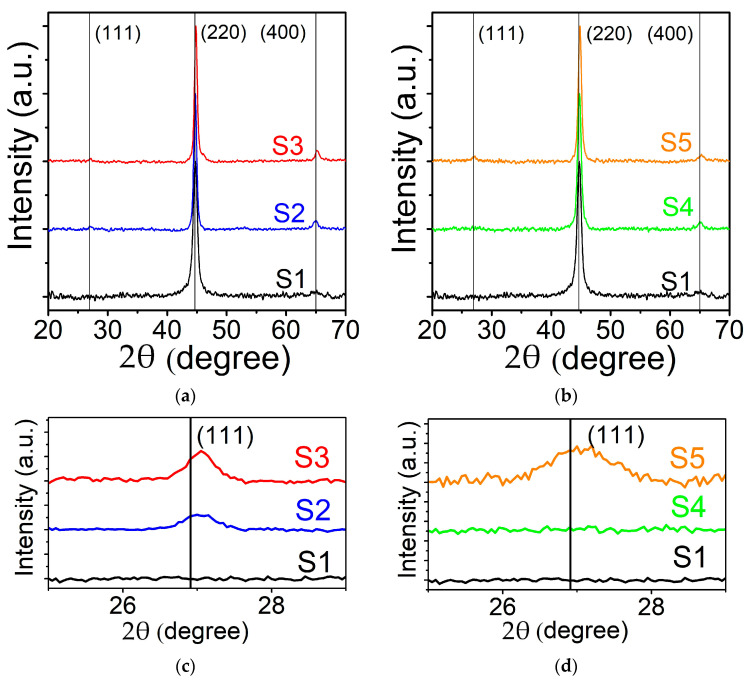
GIXRD patterns for Co_2_FeGe films: (**a**) deposited at T_S_ = RT (S1), T_S_ = 573 K (S2), T_S_ = 773 K (S3); (**b**) deposited at RT and annealed for 1 h at T_a_ = 573 K (S4), T_a_ = 773 K (S5). Close-ups for (111) reflection area for films: (**c**) deposited at different T_S_; (**d**) for films deposited at RT and annealed at different T_a_ for 1 h.

Ordered FHA, *X_2_YZ* (in our case *X* = Co, *Y* = Fe, *Z* = Ge) crystalize in the cubic *L2_1_* structure (space group no. 225, $Fm\bar{3}m$) [31]. However, different variants of disorder are observed when *X, Y* and/or *Z* atoms are intermixed. Usually for FHA thin films the following cases of disorder are considered [2]: *B2*-*Y* and *Z* atoms are randomly intermixed (space group no. 221, $Pm\bar{3}m$); *A2*-complete disorder with random intermixing (group no. 229, $Im\bar{3}m$). The most noticeable differences in the X-ray patterns for these atomically disordered phases is the absence of some superstructure reflections of the *L2_1_* type structure. Reflexes (111) and (311) are missing for both *B2* and *A2* disorders. Additionally, (200), (222) and (420) reflexes are absent for *A2* disorder. In Figure 2 GIXRD patterns are shown for the films deposited at different T_S_ (a) and deposited at RT and annealed at T_a_ (b). It is clearly seen that all the films exhibit fine polycrystalline structure. For the one deposited at T_S_ = RT only peaks (220) and (400) are observed. This is characteristic of disordered nanocrystalline film of *A2* structural type. With increasing T_S_ the width of the reflections decreases and their intensity increases. In addition, superstructure line (111) characteristic for *L2_1_* ordering appears (See close-up in Figure 2c). Similar variations are found for T_a_. However, annealing seems to be less efficient for atomic ordering comparing to deposition at elevated temperature as the superlattice reflection (111) appeared only at T_a_ = 773 K but is absent for T_a_ = 573 K (See close-up in Figure 2d). It is worth mentioning that for Co_2_FeGe the intensities of *L2_1_* and *B2* superlattice reflections are low due to nearly equal scattering factors of Co, Fe and Ge [32]. From the International Centre for Diffraction Data (ICDD) database one can find [33] for stoichiometric bulk Co_2_FeGe alloy I_111_/I_220_ ~ 0.012, I_200_/I_220_ ~ 0.0007 and I_311_/I_220_ ~ 0.0006. In our case for the samples deposited at T_S_ = 573 K and 773 K experimental value I_111_/I_220_ is in the range 0.017–0.019 that is somewhat higher comparing to theoretically calculated values. This can be due to slight Ge enrichment of our films. Reflections (200), (311) and (222) were not observed in any sample. Absence of (200) reflection is a characteristic of *B23a* structure (space group no. 227, $Fd\bar{3}m$) which represents a mix of two face centered cubic sublattices. In this structure, the first sublattice is formed with *X* atoms intermixed with *Y* atoms and the second—with *X* atoms intermixed with *Z* atoms. However, this type of atomic order is very uncommon to be experimentally realized [34]. On the other hand, significant variations in the intensities of the (200) and (311) reflections could be achieved in the case of so-called *DO_3_* or *X*-type disorders [35]. *X*-type structure (space group no. 216, $F\bar{4}3m$) is formed if the atomic number of the *Y* atom is higher than that of the *X* atom, which is not our case. For *DO_3_* space group $Fm\bar{3}m$ is conserved but *X* and *Y* or *X* and *Z* atoms are mixed on their crystallographic positions. One can suggest formation of disordered *A2* phase for the film deposited at RT. For elevated T_S_ and T_a_ a mixture of *L2_1_* and *DO_3_* phases is formed with reduced intensities of weaker reflections (200) and (311) (which fall below the detection limit of our instrument).

The evolution of lattice parameters and estimation of crystallite size determined by Scherrer’s formula [36] are presented in Table 1. Crystallite size shows expected behavior, i.e., it increases with increasing T_S_ and T_a_. The lattice parameters for the films deposited at T_S_ = RT and 573 K (a = 5.732 Å and a = 5.734 Å, respectively) correspond fairly well to the bulk stoichiometric alloy values (a = 5.738 Å) reported earlier [37,38] while for T_S_ = 773 K it is lower (a = 5.715 Å). The same tendency was found for annealed films. One can suggest that this decrease of the lattice parameter for T_S_(T_a_) = 773 K is a consequence of a compressive stress induced by differences in thermal expansion coefficients for film and Corning Glass substrate. Thus, varying T_S_ and/or T_a_ one can control formation of ordered Co_2_FeGe *L2_1_* phase, crystallite sizes and surface roughness to produce films with desired properties. In our case T_S_ = 573 K was found to be optimal to achieve the lowest surface roughness.

### 3.2. Magnetostatic Properties

**Figure 3:**
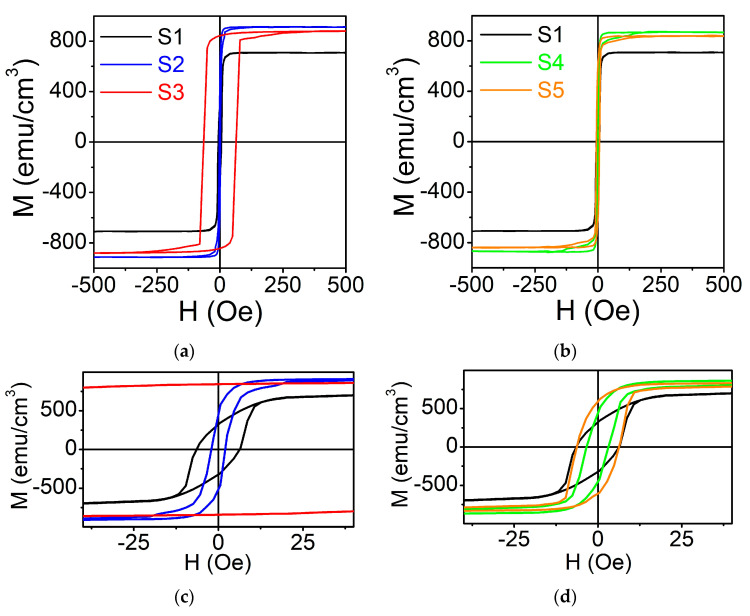
Magnetic hysteresis loops (M vs. H) for Co_2_FeGe films: (**a**) deposited at T_S_ = RT (S1), T_S_ = 573 K (S2), T_S_ = 773 K (S3); (**b**) deposited at RT and annealed for 1 h at T_a_ = 573 K (S4), T_a_ = 773 K (S5). Close-ups for −30 Oe < H < 30 Oe region for films: (**c**) deposited at different T_S_; (**d**) for films deposited at RT and annealed at different T_a_ for 1 h.

In-plane magnetic hysteresis loops measured at RT are shown in Figure 3. Determined values of saturation magnetization (*M_S_*) and coercive field (H_C_) are summarized in Table 2. Since exact volume and magnetic properties of the native oxide is not known, the values of M_S_ were calculated using nominal thickness of 60 nm for all films. All the samples demonstrate ferromagnetic behavior. The film deposited at T_S_ = RT has the smallest M_S_. The value of M_S_ first increases noticeably with T_S_ and afterwards slightly drops for T_S_ = 773 K. All the films under study, except the one deposited at T_S_ = 773 K (for which the enhanced H_C_ = 65 Oe was observed), are magnetically soft. The values of H_C_ were found to be below 6 Oe with minimum of 2 Oe for the film deposited at T_S_ = 573 K (See close-ups for −30 Oe < H < 30 Oe region in Figure 3c,d).

The variation of the magnetic properties correlates with microstructural changes. The reduced value of M_S_ for T_S_ = RT can be due to fine polycrystalline structure with *A2* type atomic disorder. The increase in M_S_ with the simultaneous decrease of H_C_ for T_S_ = 573 K can be attributed to the growth of the film with lower surface roughness and atomically ordered *L2*_1_ structure with larger crystallites. For T_S_ = 773 K the film was found to be subjected to a compressive stress (pinpointed by the reduced out-of-plane lattice parameter was observed by GIXRD) and with enhanced surface roughness. A combination of these factors could be a reason for the increase of H_C_. Similar changes in M_S_ and H_C_ with T_a_ were observed for annealed films. However, the annealing leads to smaller variation of magnetic parameters. Most probably this is because re-crystallization and formation of atomically ordered structure caused by annealing is less effective comparing to deposition at the corresponding T_S_.

The highest value of saturation magnetization M_S_ = 920 emu/cm^3^ was observed for the film deposited at T_S_ = 573 K. This is in good agreement with the results previously reported for Co_2_FeGe films in [39] while is slightly lower comparing to those from [6,22]. However, in the latter case the films were Co_2_Fe enriched. According to Slater-Pauling rule, one should expect the saturation magnetization of 6 μ_B_/f.u. (μ_B_ is Bohr magneton and f.u. is formula unit) for Co_2_FeGe FHA [2]. Considering four atoms per unit cell, experimental values of lattice parameter and applying M_S_ = 920 emu/cm^3^ one obtains M_S_~4.7 μ_B_/f.u. This value is lower than theoretically predicted and experimentally observed for bulk alloys (5.54 μ_B_/u.c.) [37] and foils (5.74 μ_B_/u.c.) [40] as well as obtained for Co_2_Fe enriched films [6]. However, our result is very similar to 4.8 μ_B_/u.c. reported for the films prepared by thermally activated intermixed reaction [41]. Reduced magnetization values per f.u. might be caused by the deviation of chemical composition from stoichiometric (i.e., slight Ge enrichment), residual atomic disorder and/or reduction of the magnetic moments on the grain boundaries of the crystallites [7,40,41,42]. It is to be pointed out that T_S_/T_a_ = 573 K seems to be optimal to produce Co_2_FeGe thin films with improved magnetic properties. This result is in a good agreement with previously published in [19,23].

### 3.3. Magnetodynamic Properties

VNA-FMR measurements were carried out in frequency domain at different applied fields $H_{ext}$. For each field value, real and imaginary parts of effective permeability parameter U(f) was calculated from the measured complex S21 spectra as described in [28]:

(1) $$U\left(f\right)=\pm \frac{i\ln\left[S_{21-H}\left(f\right)/S_{21-ref}\left(f\right)\right]}{\ln\left[S_{21-ref}\left(f\right)\right]},$$

where $S_{21-H}\left(f\right)$ denotes the set of S_21_ parameters at the FMR field of interest, and $S_{21-ref}\left(f\right)$ is the set of S_21_ parameters at the reference field. The detailed description of the method for a single resonance line analysis, the mathematical expression of the fitting function $U_{fit}\left(f\right)$ and field to frequency linewidth conversion are given in [28]. This fitting procedure provides for each peak the set of the following parameters: FMR frequency f, line width at half maximum Δf and phase shift adjustment φ.

**Figure 4:**
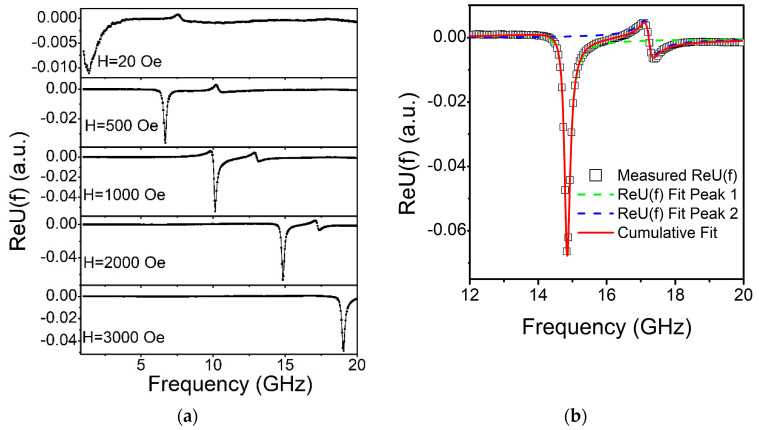
Real part of the *U(f)* function calculated from measured complex S_21_ spectrum for the film deposited at T_S_ = 573 K at different applied fields (**a**); Example of fitting of permeability Re*U(f)* for the same sample at a static applied field H_ext_ = 2000 Oe (**b**). The open circles represent the Re*U(f)* values extracted from the experimental S_21_ parameters. The two dashed lines show the best individual fitting functions Re*U_fit_(f)* for each peak while the solid curve shows cumulative fit for the whole spectrum.

The real part of the typical U(f)  spectra for different $H_{ext}\;$ is presented in Figure 4a.

For all the samples the spectra contain two resonances with visible phase shift between them. Therefore, the sum of two independent fitting functions $U_{fit}\left(f\right)$ was used to analyze the data. This approach allows one to evaluate accurately f and Δf even for the case of the vicinity of two peaks with different phases and amplitudes (see an example of the fitting in Figure 4b).

These two resonance peaks were identified as uniform FMR precession (the more intense peak) and first Perpendicular Standing Spin Wave (PSSW) modes. Analysis of the frequency dependence of the main peak on applied magnetic field allows estimation of the effective magnetization $M_{eff}$ of the sample using Kittel’s formula [43]:

(2) $$f_{FMR}=\frac{\gamma }{2\pi }\sqrt{\left(H_{ext}+H_{ani}\right)\left(H_{ext}+H_{ani}+4\pi M_{eff}\right)}$$

where $f_{FMR}$ is the FMR resonance frequency, γ is the gyromagnetic ratio, $H_{ext}$ and $H_{ani}$ are the applied magnetic field and uniaxial in-plane anisotropy field, respectively. It is to be noted that $M_{eff}$ differs from M_S_ determined from magnetostatic SQUID measurements. The expression for $M_{eff}$ is given in [44] as $4\pi M_{eff}=4\pi M_{S}-H_{⊥}$, where $H_{⊥}$ is a sum of all possible perpendicular anisotropy fields—magnetoelastic, magnetocrystalline and Neel surface anisotropy. Apart perpendicular magnetic anisotropy, additional difference between $M_{S}$ and $M_{eff}$ can arise due to the fact that static SQUID measurements are magnetic-volume dependent, while FMR ones are independent. If sufficient volume of nonmagnetic material is present in the sample (like thick magnetically dead oxide layer on the surfase), experimentally determined $M_{eff}>M_{S}$.

Frequency dependence of PSSW was used to extract the exchange stiffness constant A. In [45] $f_{PSSW}$ was given as:

(3) $$f_{PSSW}=\frac{\gamma }{2\pi }{\left[\begin{array}{c} \left(H_{ext}+H_{ani}+\frac{2A}{M_{eff}}{\left(\frac{p\pi }{d}\right)}^{2}\right)\times \\ \times \left(H_{ext}+H_{ani}+\frac{2A}{M_{eff}}{\left(\frac{p\pi }{d}\right)}^{2}+4\pi M_{eff}\right) \end{array}\right]}^{1/2}$$

where, p is the order of PSSW (*p* = 1 in our case) and d is the thickness of the film.

**Figure 5:**
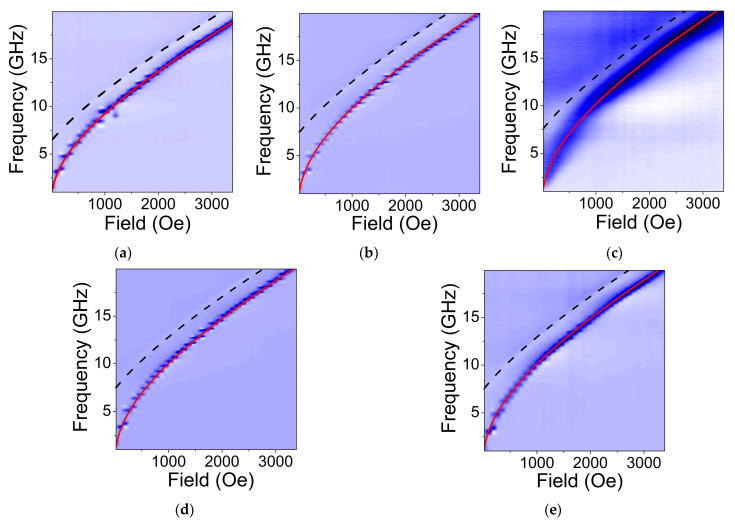
Dependencies of the FMR frequency and the first order PSSW on applied magnetic field for Co_2_FeGe films deposited at various conditions: (**a**) T_S_ = RT; (**b**) T_S_ = 573 K; (**c**) T_S_ = 773 K; (**d**) T_S_ = RT+ annealing at T_a_ = 573 K for 1 h; (**e**) T_S_ = RT+ annealing at T_a_ = 773 K for 1 h. Solid red lines represent fit of the experimental data for FMR using Equation (2). Dashed lines represent fit for the first order PSSW using Equation (3). The values of effective magnetization $M_{eff}$ and the exchange stiffness constant A extracted from the fitting are listed in Table 2.

Dependence of the FMR frequency and the first order PSSW vs. $H_{ext}$ are shown in Figure 5. Lines represent fit of the experimental data for $f_{FMR}$ and $f_{PSSW}$ using Equations (2) and (3), respectively. Estimated values of $M_{eff}$ and A are presented in Table 2. The in-plane anisotropy $H_{ani}$ extracted using Equation (2) was found to be vanishing (<10 Oe for all cases). It is in a good agreement with prediction that for fine polycrystalline films (like ours) $H_{ani}$ can be neglected due to the randomization of the magnetocrystalline anisotropy axis within sample volume [17]. The gyromagnetic ratio γ/2π shows no significant variation both with T_S_ and T_a_. The estimated values are within 2.86–2.88 MHz/Oe. These correspond to the g-factor of 2.04–2.06 that point on weak spin-orbit interactions and correlates well with the earlier reports on Co_2_FeGe films of different compositions [5].

Significant linewidth broadening was observed for the film deposited at T_S_ = 773 K. For this case the magnetic parameters were evaluated from FMR measurements with higher uncertainty in comparison with the other films. For all films, apart the one deposited at T_S_ = 773 K, the values of $M_{eff}$ determined by FMR correlates within experimental error with M_S_ from static SQUID measurements. This indicates that no significant out-of-plane anisotropies are presented in the films under study. Exchange stiffness constant for as-deposited films is relatively low A ~ 6.8 pJ/m. Both elevated T_S_ and annealing lead to the rise of A. However, the highest achieved values A ~ 9−10 pJ/m are still lower than for epitaxial Co_1.5_Fe_1.5_Ge films (A=13 pJ/m) [22]. It is well known [46] that the decrease of the exchange constant is associated with dilution magnetic atoms by non-magnetic. In our case for as deposited films Ge atoms are randomly placed within the crystal lattice leading to effective reduction of A. Elevated T_S_ and annealing lead to the improved atomic ordering and to the consequent increase of exchange stiffness, although it does not reach the values of epitaxial Co_2_FeGe films with well-ordered structure. In addition, from EDAX data the films show an excess of Ge. This also can explain the reduction of *A* as the proportion of non-magnetic atoms is higher. One should keep in mind that the estimation of A in our case is relatively rough. First, the thickness of the films d=60 nm was used for the estimation. The ‘dead’ magnetic layer associated with native surface oxide and crystallite boundaries in nanostructured films were not considered in the calculations because the estimation of their actual ratio is speculative. Second, only the 1st order standing spin wave was observed in the experiment leading to limitations in applicability of the Equation (3) [47].

Finally, Gilbert damping parameter α can be determined from FMR linewidth ΔH using the formula [28]:

(4) $$\Delta H=\Delta H_{0}+\frac{4\pi \alpha f}{\gamma }$$

where $\Delta H_{0}$ is a measure of inhomogeneous broadening related to films quality. It is to be pointed out that linewidth broadening generally contains contributions from the intrinsic (Gilbert damping) and extrinsic (two-magnon scattering, inhomogeneity of magnetic properties in the measured material, angular dispersion of crystallite orientation) mechanisms.

**Figure 6:**
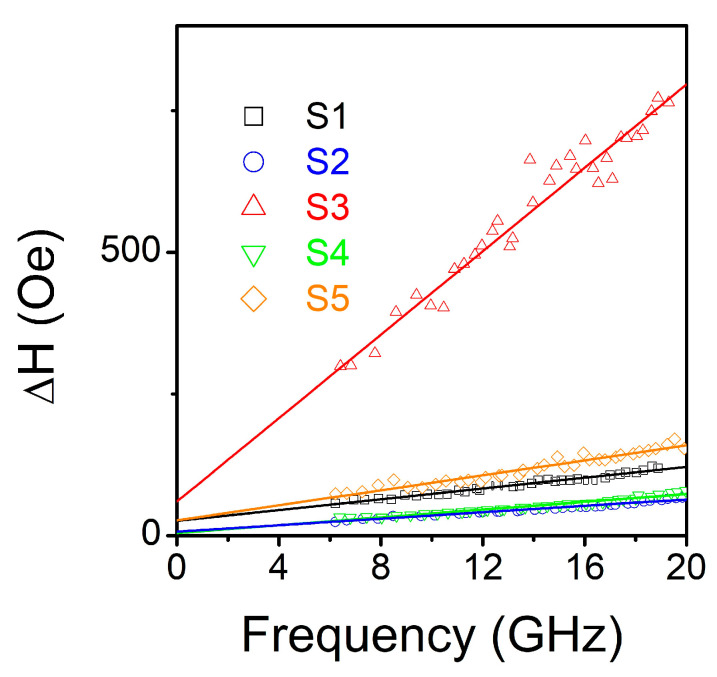
Dependencies of FMR linewidth ΔH as a function of resonance frequency for Co_2_FeGe films deposited at T_S_ = RT (S1), T_S_ = 573 K (S2), T_S_ = 773 K (S3) and deposited at RT and annealed for 1 h at T_a_ = 573 K (S4), T_a_ = 773 K (S5). Experimental data (points) are accompanied by fit (lines) according to Equation (3). Estimated values of damping parameter α are listed in Table 2.

Previously it was shown that for CoFeGe films with thickness above 50 nm [5] contribution from two magnon scattering [48] is negligible. It was mentioned in [49] that two-magnon scattering can add non-linear contribution to the ΔH(f) dependence that was not observed in our measurements. Indeed, our experimental data is fitted fairly well using linear dependence, Equation (4). Fitting results are presented in Figure 6 and estimated values of α listed in Table 2.

Damping parameter α depends non-monotonically on both T_S_ and T_a_. It is already relatively low for as deposited films (~0.007) and first decrease with T_S_ (or T_a_). The minimal value of α ~ 0.004 was observed for the film deposited at T_S_ = 573 K, and similar α~0.005 was obtained for T_a_ = 573 K. These values are consistent with reported earlier for CoFe-Ge films, although do not reach the lower limit [5,19,22] ~0.0025. This can be explained by the difference in microstructure of the films under investigation. Films in the abovementioned works were grown epitaxially on MgO single-crystalline substrates, while ours are polycrystalline. It is known [50] that grain boundaries cause non-uniformity in the internal magnetizing field or in demagnetizing field due to the internal defect. Another possible reason is that the chemical composition of our films (in particular, CoFe alloy component) is different from the one in the citing references. However, the values of α obtained in this work are smaller than those reported earlier for epitaxial Co_2_Fe(Ge_0.5_Ga_0.5_) [51], widely used CoFeB [52] and some Co_2_FeSi [18] films.

The further increase of processing temperature leads to the increase of α, slight in the case of annealing, or drastic (α ~ 0.06) for T_S_ = 773 K. In these cases, the films are characterized by reduced lattice parameter with respect to bulk Co_2_FeGe phase. In addition, this film shows higher surface roughness. A noticeable increase of damping was reported for Co_2_FeSi films annealed at the elevated temperatures [53]. It was attributed to the influence of Cr buffer and atomic intermixture on Cr/Co_2_FeSi interface. In our case no buffer or capping layers were used. Thus, additional contributions to damping associated with variation of chemical composition, tense stress, formation of textured structure and surface effects [18,54] should be considered.

From magnetic measurements one can evaluate the exchange length $l_{ex}$, an important parameter that dictates magnetization reversal [55]. It is essential for practical applications and micromagnetic simulations and can be described as the distance over which the perturbation due to the switching of a single spin decays in a soft magnetic material [56]. In [55] it was given as lex=A2πMS2. Using values from Table 2 one can get $l_{ex}\;\sim \;4.6\;$nm for the film deposited at T_S_ = RT and $l_{ex}\;\sim \;4.2\;$nm for the film deposited at T_S_ = 573 K. The case of T_S_ = 773 K was not evaluated here because of big experimental error in determination of A associated with broadening of the FMR line. The annealing does not lead to significant changes in $l_{ex}$. The values of $l_{ex}$ determined here are slightly lower comparing to those of CoFeB [52] and Co_1.5_Fe_1.5_Ge [22] films and are typical for soft magnetic materials [55].

It is interesting to note that according to the data reported in [19], annealing at T_a_ = 673 K leads to the increase of damping for Fe_1.5_CoGe films, while for T_a_ = 773 K no visible FMR peak was found, suggesting the degradation of magnetic properties with increased T_a_. Contrary to this, broadened FMR resonance peaks are still visible for our films deposited or annealed at 773 K. Simultaneously, slight decrease in M_S_ and strong increase of H_C_ and α were observed at T_S_ = 773 K. Thus, one can conclude that the growth conditions associated with T_S_ = 773 K cause a degrading effect on dynamic magnetic properties of the Co_2_FeGe films. A suggestion can be made that the effect of a compressive stress or formation of the film with strong contribution from crystalline anisotropy is a cause for such behavior. Physical mechanisms associated with these variations should be studied carefully in the future works.

## 4. Conclusions

Polycrystalline Co_2_FeGe Heusler alloy films with close to stoichiometry composition were fabricated using DC-magnetron co-sputtering technique from Co_2_Fe and Ge targets. Influence of deposition conditions, namely, substrate temperature and post-deposition annealing on magnetic properties was studied. Elevated substrate temperature and annealing promote formation of ordered L2_1_ phase. Damping parameter α as low as 0.004 was achieved. This value is consistent with predicted for half-metallic compounds having a gap in minority density of states on Fermi level. It was found that saturation magnetization and exchange stiffness constant for the investigated films are relatively low. Our study demonstrates that the crystal structure, grain size and roughness can be controlled by adequate growth conditions and consequently, both static and dynamic magnetic properties could be effectively tuned in a wide range using these parameters. This paves the way to a possible future technological application of these films as some of them present the proper magnetic properties (low damping parameter, saturation magnetization and exchange stiffness) for spin-transfer torque devices with reduced operation current.

## Figures and Tables

**Table 1:** Structural parameters obtained from XRR and GIXRD in all the samples as a function of the temperature used for deposition (T_S_) and annealing (T_a_). The film thickness (*t*), surface roughness (Δ*t*) and density (*ρ*) were determined from the fits to the experimental XRR patterns with a chi-square value below 3 × 10^−2^. The lattice parameter (a) and crystallite size (d) were calculated from GIXRD within ±0.002 Å and ±0.5 nm accuracy, respectively.

T_S_, K	T_a_, K	From XRR			From GIXRD	
		*t*, nm	Δ*t*, nm	*ρ,* gr/cm^3^	a, Å	d, nm
RT	---	57	0.63	8.3	5.732	10
573	---	61	0.47	8.64	5.734	14
773	---	61	2.1	8.66	5.715	15
RT	573	59	0.63	8.31	5.732	11
RT	773	57	0.63	8.69	5.713	13

**Table 2:** Deposition temperature (T_S_), temperature of annealing (T_a_), saturation magnetization (M_S_) and coercive field (H_C_) determined from SQUID measurements, effective magnetization (M_eff_), exchange stiffness (A) and damping parameter (α) determined from FMR measurements. Error margins for M_S_ were estimated from uncertainty of the sample size determination. Error margins for FMR measurements were derived from fitting procedure.

T_S_, K	T_a_, K	From SQUID		From FMR		
		M_S_, Emu/cm^3^	H_C_, Oe	M_eff_, Emu/cm^3^	A, pJ/m	α
RT	---	710 ± 35	6 ± 1	738 ± 10	6.8 ± 0.2	0.007 ± 1.5 × 10^−4^
573	---	920 ± 50	2 ± 0.5	895 ± 10	9.2 ± 0.3	0.004 ± 1.1 × 10^−4^
773	---	880 ± 45	65 ± 1	930 ± 180	9.3 ± 1.2	0.06 ± 6 × 10^−3^
RT	573	870 ± 45	3 ± 0.5	906 ± 10	9.8 ± 0.3	0.005 ± 1.7 × 10^−4^
RT	773	840 ± 45	6 ± 1	882 ± 40	9.4 ± 0.3	0.009 ± 6 × 10^−4^

## Data Availability

The data presented in this study are available on request from the corresponding author.
